# Post-Mortem

**Published:** 1872-10

**Authors:** W. H. Coe, Jas. B. Baird

**Affiliations:** Atlanta, Ga.; Atlanta, Ga.


### POST-MORTEM.

REPORTED BY W. H. COE, M.D., AND JAS. B. BAIRD, M.D., ATLANTA, GA.

Adam ——, colored, a day laborer, aged twenty-one years, single, had an attack of acute pleuritis, in the left side, last March, from which he made a good recovery, and considered himself well.

On the 9th of September last, he complained of a feeling of malaise and general debility; this continued until the evening of the 12th, when he took to his bed, complaining of a slight pain in the region of his heart and in his head. Pulse one hundred and twenty per minute; skin hot and dry.

At seven o'clock next morning, pulse one hundred and forty-five per minute—weak and irregular; active delirium; pupils dilated; urine passed involuntarily.

At eleven o'clock same morning, pulse one hundred and thirty, regular; skin cool, covered with sweat; respirations forty per minute; sensation and motion not impaired; no muscular rigidity—no heat about the head; otherwise, condition unchanged. A clear and loud friction sound was heard over the præcordia, corresponding accurately with the systole and diastole of the heart, and limited directly to the præcordial space, having abrupt boundary lines on every side.

The diagnosis, based upon the physical signs, was acute pericarditis; and in view of the infrequency of acute idiopathic pericarditis, and from the fact that the patient had had several attacks of acute articular rheumatism, the opinion was ventured, that the pericarditis was dependent upon the rheumatic diathesis, and that rheumatism would develop itself in a few hours. The nervous symptoms were not more marked than is frequently the case in severe attacks of acute pericarditis, and which frequently mislead the physician, causing him to look to the brain for the origin of the disturbance. The treatment consisted in the use of cardiac sedatives, counter-irritation over the præcordia, enemata, and an occasional stimulant to counteract the depressing influence of the sedative. The patient grew rapidly weaker, and died at nine o'clock on the morning of the 14th.

*Autopsy, Five Hours after Death.*—Lungs healthy; pericardium white, glistening—not at all injected; contained about four drachms of fluid—clear, not a flake of lymph to be found; surfaces perfectly smooth.

How, then, could that *pericardial friction sound* be accounted for? The cause was soon discovered. Already, in attempting to pass the hand between the left lung and the pericardium, adhesions so extensive and so firm had been encountered, that it was necessary to dissect the pulmonary pleura from the pericardium with the scalpel. There was also an adhesion, about the size of a silver half-dollar, between the costal and pulmonary pleura, just under the left nipple, and the surfaces of the pleura were very much roughened by the deposit of lymph over the entire præcordia. This unusual condition was due, no doubt, to the attack of acute pleuritis from which the patient suffered last spring.

Thus we see, as the lung was closely bound to the pericardium, and the two layers of the pleura roughened and adherent, that, at every movement of the heart, the rough surfaces of the pleura were brought in contact, and rubbed together, and a sound produced which it was impossible to distinguish from the pericardial friction murmur.

We regret that our time did not permit us to examine the brain, as the cause of death would, no doubt, have been found there.

Feeling that the results of this post-mortem examination may be of service to others, we are constrained to make them public.

